# Ethical Diversity and the Role of Conscience in Clinical Medicine

**DOI:** 10.1155/2013/587541

**Published:** 2013-12-12

**Authors:** Stephen J. Genuis, Chris Lipp

**Affiliations:** ^1^University of Alberta, 2935-66 Street, Edmonton, AB, Canada T6K 4C1; ^2^University of British Columbia, 2329 W Mall, Vancouver, BC, Canada V6T 1Z4

## Abstract

In a climate of plurality about the concept of what is “good,” one of the most daunting challenges facing contemporary medicine is the provision of medical care within the mosaic of ethical diversity. Juxtaposed with escalating scientific knowledge and clinical prowess has been the concomitant erosion of unity of thought in medical ethics. With innumerable technologies now available in the armamentarium of healthcare, combined with escalating realities of financial constraints, cultural differences, moral divergence, and ideological divides among stakeholders, medical professionals and their patients are increasingly faced with ethical quandaries when making medical decisions. Amidst the plurality of values, ethical collision arises when the values of individual health professionals are dissonant with the expressed requests of patients, the common practice amongst colleagues, or the directives from regulatory and political authorities. In addition, concern is increasing among some medical practitioners due to mounting attempts by certain groups to curtail freedom of independent conscience—by preventing medical professionals from doing what to them is apparently good, or by compelling practitioners to do what they, in conscience, deem to be evil. This paper and the case study presented will explore issues related to freedom of conscience and consider practical approaches to ethical collision in clinical medicine.

## 1. Introduction

The practice of contemporary medicine is changing. With diverging views about what constitutes acceptable and professional behavior, one of the most formidable tasks facing the medical community is how to respond to ethical diversity within its membership. Issues of conscience are becoming increasingly problematic for healthcare personnel as nurses, physicians, and other members of the healthcare team endeavor to interact with the expanse of emerging medical technologies, and to respond to evolving expectations that involve more than just treating disease and alleviating suffering [1]. When making clinical decisions, physicians are now tasked with balancing diverse priorities such as promoting wellness, conserving resources, measuring up to continuously evolving standards, making decisions about quality-of-life, engaging in advocacy, and changing harmful patient behaviours [2].

Furthermore, juxtaposed with waning respect for the wisdom of individual conscience and personal ethical conviction, pressure from sources external to clinical healthcare (including some lawyers, bioethicists, and politicians) is now being exerted on medical professionals to unquestioningly act in allegiance with peer standards and professional governance. While acting in good conscience represents the essence of individual integrity for some practitioners, going “against the flow” due to conscientious or ethical conviction is increasingly portrayed as “unprofessional” and disparagingly depicted as acting according to personal preference. There is uncertainty as to whether escalating ethical diversity within contemporary medicine is an asset or a liability to cohesion with the medical community and to the provision of optimal clinical healthcare.

Amidst the emerging landscape of diverse and often conflicting ethical perspectives, this paper will (i) briefly address the concept and the role of personal conscience; (ii) survey the existing literature on conscience-related issues in healthcare; (iii) describe dichotomous perspectives on the installation of measures to secure “freedom of conscience;” (iv) explore practical workplace issues and approaches for health providers; (v) advance benefits and risks of conscience rights for health professionals; and (vi) provide a case study highlighting some of the challenges associated with making a dissenting conscience decision.

Fundamental guiding questions for this paper include the following.

- When health providers disagree with their patients, colleagues, or regulatory professional bodies about the suitability of specific types of care, what standard should provide a point of reference for the practitioners' ethical course of action?
- Is it acceptable to punish health providers (professional discipline, loss of privileges, loss of job, etc.) because of their commitment to act in accordance with their firmly held ethical position?
- What impact does acquiescence to regulatory edicts have on health professionals who hold ethical or moral reservations about existing clinical standards/guidelines?

### 1.1. What Is Conscience?

The Greek etymology of conscience literally means “with knowledge” [3]. The Oxford dictionary describes conscience as “a person's moral sense of right and wrong, viewed as acting as a guide to one's behaviour” [4]. Thus, conscience may be simply understood as a metaphysical guide that acts in a judicial way to direct a person's actions. In day-to-day living, conscience seems to be closely related to a person's beliefs or convictions about actions that are deemed morally right or wrong [1].

Despite the prevalence and fervor with which conscience issues are explored in medical writing [5–7], a clear definition of conscience in healthcare settings is lacking. In the medical literature exploring conscience issues, few authors explicitly define terms. Some medical ethicists, however, consider conscience as having two main components. First, a person's conscience is rooted in a fundamental responsibility to consider all situations within a framework of ethical obligation [8]. Second, this responsibility leads to judgments and reasoning about the types of actions and behaviours which characterize a moral life [1]. Rather than the reductionist perspective that conscience is a mystical intuition based on emotions, feelings, or preferences, conscience represents the decision-making capacity of the human mind founded on a desire to live an upstanding and honourable life which promotes good for oneself and for others [1].

Authors exploring the notion of conscience use a variety of terms to characterize the multidimensional role of conscience in one's life [1, 9–11]. Conscience has been described in its role as a means to preserve integrity or ethical wholeness (“perfective conscience”) [12], and is used to monitor how potential decisions resonate with, or “protect” one's moral framework. Other authors describe the role of conscience both retrospectively (looking back on previously made decisions or actions) and prospectively (assessing whether a proposed action would compromise one's moral integrity) [1]. Human conscience, most succinctly described, seems to involve a moral decision-making faculty, influenced by a rational perception of the observable world which is both reflective and reflexive [10]. The reflective nature of conscience scrutinizes past, present, and future decisions, while the reflexive component provides instant feedback in the form of internal dissonance or discomfort when an individual is compelled to choose a potentially problematic or immoral decision or action.

### 1.2. Present-Day Ethical and Conscience Dilemmas in Healthcare

Dilemmas of conscience in medicine are increasingly encountered by healthcare providers from a spectrum of clinical disciplines. From our survey of the literature as well as through personal experience, a few examples of the myriad situations that involve ethical consideration with patients, peers, or regulators are presented (Table 1).

## 2. Background on Issues of Conscience in Healthcare

The vast and expanding scope of medical practice combined with increasing diversity of opinion within modern society has led to escalating public discussion of conscience issues in healthcare [6, 27–29]. Various terms including “moral stress” [30], “moral distress,” and “ethical distress” [31] have been used to describe the existential anguish experienced by health professionals when facing challenging ethical situations. In the academic and grey literature, the majority of conscience issues are discussed somewhat imprecisely within two general domains: (1) stress of conscience and (2) freedom of conscience.

### 2.1. Stress of Conscience

The majority of research relating to stress of conscience refers to situations where health providers are unable to fully address the needs or challenges of those receiving care [32, 33]. These factors may lead to a “troubled conscience” [11], or “stress of conscience” [32] among practitioners as a consequence of failure to attain what their conscience expects or demands of them to do [33].

Analyses of the impact of conscience stress within healthcare settings [34, 35] have generally tended to focus on outcomes for healthcare systems and patient recipients rather than for medical providers. However, some nursing research has been done through validated questionnaires in fields including psychiatry [36, 37], geriatric care [38–41], neonatal nursing [42], and intensive care [43–45]. These surveys assess personnel perception of conscience [33], stress of conscience [32, 34, 35], and the impact of ethical stressors on healthcare providers and on patient care [32, 33]. The research consistently supports the observation that elevated stress of conscience is a contributor to nursing burnout [32, 34–36, 40, 46], job dissatisfaction [41], and the provision of suboptimal patient care [36].

Although the impact of stress of conscience among physicians is inadequately researched, there are some preliminary studies which document moral distress and the associated burden of anguish resulting from certain ethical situations among clinicians in nephrology [47], podiatry [48], general medicine [49], and critical care medicine [50, 51]. Research confirming stress of conscience has also been conducted among medical students and residents [52–54] indicating the commonality of this experience during medical training. Long-term sequelae of sustained or repetitive conscience stress in physicians and medical trainees have not been sufficiently investigated to date. Anecdotally, many physicians find the increasing prevalence of ethically challenging situations to be an unwelcome burden, with some practitioners modifying their professional duties or leaving positions to avoid such encounters. Some practitioners avoid serious ethical decision-making by referring to, and abiding by, the dictates of designated ethical experts such as ethicists or ethics committees.

### 2.2. Freedom of Conscience

The second context where conscience issues arise involves direct situations of ethical collision; in these situations a healthcare provider is asked or expected to participate in a specific action he or she deems to be ethically wrong. This second connotation of the expression “conscience issues” evokes phrases such as “freedom of conscience” (FC), “conscientious objection,” “conscience rights,” and “conscience clauses” [5, 10, 27, 55–57] along with moral and ethical distress. The remainder of this paper will focus on exploring issues related to FC (freedom of conscience).

Political, legal, and legislative events in recent decades have brought conscience issues to the forefront. Not only have well-known politicians discussed the issue of conscience legislation in election platforms [28, 29], but legal and legislative bodies have begun to pass judgments on this issue. For example, in a recent ruling from the College of Physicians and Surgeons of Ontario (CPSO), physicians were clearly warned that they could be found in violation of the Ontario Human Rights Code if, based on moral or religious beliefs, they refused to provide a service to a patient [58].

This type of authoritarian approach to conscience rights has begun to be implemented in various jurisdictions and domains. For example, financial penalties and/or imprisonment exist for health providers who act contrary to public policy in the Philippines [59, 60], and a proposed ruling by the US Department of Health and Human Service would enforce employers to pay for employees' contraception regardless of employers' moral or religious objections [61]. Yet, while issues of conscientious objection are engendering greater significance in political and legal proceedings [62–64], little attention has been applied to understanding how enforced restriction of conscience rights might affect individuals navigating situations of ethical collision, and specifically to understanding the short- and long-term impacts of coerced complicity in healthcare settings.

A number of surveys have been conducted to determine sentiment and support for the principle of FC in healthcare settings [6, 7]. While medical students and nurses have been polled on this matter [65, 66], the broadest discussion has come from clinicians and bioethicists who have theorized about and explored both the importance of conscience rights [10, 67, 68] and the associated hazards of such rights [27, 55, 57]. The perspectives vary considerably.

### 2.3. The Polarizing Status of Conscience Matters in Medicine

The intense debate about the benefits and hazards of securing conscience rights highlights a strong polarity within the healthcare community. On one hand, some physicians, ethicists, policy-makers, and lawyers adamantly object to FC legislation and argue that every physician should be professionally required to carry out legal medical services at a patient's request, regardless of the physician's ethical convictions or religious beliefs [55–57, 69–76]. On the other hand, supporters of conscience rights argue that absolute regulation requiring professionals to be willing to act contrary to their own personal values is imprudent, prejudicial, and unacceptable.

#### 2.3.1. Opposition to Freedom of Conscience Legislation

Those opposed to a sweeping policy to secure FC rights contend that such liberty erodes patient autonomy and the societal role or professional obligations of the physician [56, 69, 76]. Many ethicists and lawyers argue that conscience clauses lead to dysfunctional healthcare delivery and compromise the quality of patient care [55, 57, 70–75]. Other arguments against FC legislation include the assertion that no patient should ever be obstructed from receiving legal medical care based solely on a physician's personal values [77]. Not only would this obstruction violate patients' autonomy in choosing the type of health care services they deem most appropriate to their own needs [71], but FC opponents also contend that this level of legislation regresses medicine into a paternalistic system where the doctor is the ultimate decision-maker rather than the patient [57, 78].

In addition, it has been contended that FC promotes an attitude of unprofessionalism amongst those who take advantage of the freedom and privilege it offers [57]. It is suggested that FC legislation may encourage physicians to provide healthcare based solely on individual preferences or whims, rather than broader public interests. Immanuel Kant's universal applicability principle argues that there is only a single categorical imperative, which is to “act only in such a way that you can will that the maxim of your actions should become a universal law” [79]. Kant's contention is that broader public interest should trump individual preference; he proposes that it is better for one person to experience internal friction than for the whole state to be disrupted. A study of this ideology has led some analysts to conclude that physicians should divorce themselves from their conscience and beliefs about what is good and right, and execute their duties as “neutral arbiters of medical care” [71].

Furthermore, from a practical standpoint, FC legislation would seemingly complicate the healthcare system and compromise any united standard of care [74]. Patients who request or require urgent care could be refused assistance by physicians maintaining a conscientious conviction against such type of care, and thus hospital administrators and patients would have to search for other health providers to meet patients' needs [57]. In addition, it is alleged by some that once religious and moral objections significantly affect medical care, society will be impaired in its ability to make science-based decisions and informed social progress, an example of such an allegation is the current move in many jurisdictions by those with moral misgivings, to obstruct the legal incorporation of physician-assisted death [76].

It is also assumed by many FC opponents that conscientious conviction usually represents religious affiliation, and thus they assert that religious edicts and influences have no claim in the marketplace of secular healthcare [57, 69, 80]. Dogmatic admonitions highlighting this position have been issued recently; for example, an edict by a state human rights body warned that doctors, as providers of services that are not religious in nature, must essentially “check their personal views at the door” in providing medical care [81]. The American College of Obstetrics and Gynecology also provided the recommendation recently that “Conscientious refusals should be limited if they constitute an imposition of religious or moral beliefs on patients” [82].

Some ardent adherents of this perspective also state that physicians who refuse to comply with legally accepted and established medical treatments are not qualified to fulfill the role of a professional within the medical community, and should therefore be asked to find a more suitable profession or medical specialty with no threat of conscience dilemmas [27, 55, 57, 71]. For example, an article in a prominent Canadian medical journal asserts: “Physicians who feel entitled to subordinate their patient's desire for well-being to the service of their own personal morality or conscience should not practice clinical medicine” [83].

#### 2.3.2. Support for Freedom of Conscience Legislation

Individuals and groups representing the other side of the debate raise various issues and provide refutations. Many physicians, philosophers, and medical trainees are in full support of FC for health professionals [5, 18, 68, 84], arguing that preserving conscience rights is in the best interests of healthcare providers, patients, and society. Some interpret Kantian-based philosophy to suggest that if successive physicians lose individual liberty of conscience and are morally compromised because of authoritarian dictates, the end result will be a diminishing of collective professionalism and physician morale, leading to inadequate patient care [22].

Proponents of FC advocate that ethical decisions are pervasive in clinical medicine, and that making conscience-based decisions goes far beyond personal preference and represents the essence of what health providers actually believe is best for patients [85]. It is argued from this vantage point that physicians who hold to their conscience values when faced with ethical distress maintain personal integrity and moral sensitivity, thus fostering a culture of respectful consideration which promotes patient well-being and furthers ethically-cognizant medical advancement [10, 86]. Trespassing the bounds of personal conscience, they contend, results in severe compromise to individual self-respect, integrity, and personal job satisfaction [10, 87]—qualities integral to physician well-being.

Conscience supporters generally reject the notion of a distinctive “professional conscience” separate from a “personal conscience.” Rather, practitioners are deemed to have only one conscience; those in favour of FC assert that the notion of maintaining modifiable contradictory values depending on circumstances defies the definition of “conscience.” Philosophic literature is also used by FC advocates to add credence to their arguments. Contemporary moral philosopher Alasdair Macintyre contends that “encouraging physicians to separate themselves and their values from the roles they perform, is a recipe for the dissolution of character” [88]. Compromise of personal moral integrity, of any kind or nature, will inevitably lead to an erosion of ethical behaviour—a prospect not conducive to optimal provision of healthcare [86].

In addition, some argue that conscience provides an invaluable intrinsic checkpoint in urgent ethical dilemmas [10]. This checkpoint serves as an indispensable aid to practitioners facing acute care dilemmas in the intensive care unit or emergency department. When confronting a pressing ethical dilemma requiring immediate decision-making; for example, a physician may have little else to turn to other than conscience.

While antagonists of FC may argue that conscience is an impediment to patient-centred values and patient experience [57, 69], proponents argue otherwise. Supporters often contend that FC promotes open, transparent physician-patient relationships and engenders patient advocacy and trust. At the core of an attitude of advocacy for patients is the physician-patient relationship and unadulterated trust in the caregiver. Fundamental patient-centered values include honesty, faith that the caregiver will always act ethically and do what is best for the patient, and security that the clinician will never agree to covertly harming the patient. Physicians who possess self-awareness of their own values and beliefs are able to recognize and communicate their own biases [86]. This open communication fosters honesty and allows patients to objectively decide whether their physician is a trustworthy and competent practitioner who is able to provide high-quality health care services. It is unlikely that individual patients or society would support a situation in which physicians were being coerced to hide their convictions, making decisions they felt were morally wrong or unethical, or failing to act in what they perceived to be their patients' best interests.

It is claimed that FC also facilitates public advocacy for disadvantaged individuals and groups [10]. Public advocacy generally involves personal risk to the advocates as they are resisting the status quo and often contending against vested interests that are alleged to be subversively harmful to patient and societal wellbeing. With allegations of physician intimidation in some jurisdictions [89], protection of conscience rights permits a culture of advocacy in which health providers are given the liberty to be patient advocates in defiance of authoritarian dictates. A recent public event serves to illustrate unavoidable consequences of removing FC rights; a conscientious physician was severely reprimanded by authorities for speaking out against industrial practices he claimed were harming the environment and endangering the health of a local community [90]. While it has been alleged that conscience-based physicians are simply serving their own personal interests, those acting from a perspective of deeply-seated conscience conviction often manifest considerable courage and honorable intention as they serve others and sometimes endure personal risk.

Preserving FC promotes the physician as an independent, objective, and autonomous caregiver rather than an instrument of the state [87]. History is rife with instances where delivery of independent, ethical medical care was compromised with disastrous results. The atrocities committed by Nazi physicians and, more recently, those of some American physicians working in Iraq and Afghanistan are testaments to the potential brutal activity that can occur when governments stifle the consciences of physicians [68]. Furthermore, humanity suffers when physicians become silent soldiers marching to the beating drum of an oppressive regime [91]. A widespread dismissal of conscience socializes physicians to be muted participants in atrocities and suboptimal care rather than advocates of health and humanity [68]. While this sort of regime seems foreign to North American medicine, physicians are increasingly facing less and less emphasis on good care and virtuous behavior [86, 87] and more emphasis on adhering to external guideline panels.

As the practice of medicine necessarily involves the incorporation of morals and ethics, varying interpretations of values should be expected and tolerated within any diverse group of professionals [87]. Even a misguided conscientious objection may demonstrate ethical leadership and integrity [86]. Furthermore, advocates for conscience rights often remind critics that modern medicine allegedly encourages the critical analysis of status quo ideas and practices through thoughtful reason and ingenuity. In fact, the major historical advances in medicine throughout time have unfailingly been the result of thoughtful dissonance and the challenging of existing practices in an attempt to change course [22]. Encouraging ingenuity, critique, and creativity yet squashing nonconformity is argued by FC advocates to be oxymoronic.

Furthermore, many objections to specific interventions and the corresponding desire to secure FC are based on issues of quality of care, scientific credibility, human rights, environmental implications, and preservation of dignity rather than exclusively religious or ideological rationale. To illustrate this, it is important to understand the concept of “standard of care” (SOC). Individual physician behavior is often measured against the grid of clinical practice guidelines, which are medical practice directives delineating the SOC to guide physicians about what is expected in specific clinical situations. It is often assumed that SOC proclamations and clinical practice guidelines represent informed science, cutting edge research, and up-to-date information in the scientific realm. The reality, however, is that knowledge translation in science is notoriously slow and SOC provisions are often influenced by agenda-driven vested interests and are often out of date with what emerging research is demonstrating [92–96]. This lethargy of knowledge translation prompted a Nobel Prize winner to comment: “A new scientific truth does not triumph by convincing its opponents and making them see the light, but rather because its opponents eventually die, and a new generation grows up that is familiar with it.” [97] In fact, conscientious individuals may express their iconoclastic views because they perceive that a strong stand is required against vested interests, against harmful interventions, and against entrenched patterns of misguided practices that are not in the best interests of patients, the medical profession, or society as a whole.

Finally, there exists an allegation that certain vocal opponents of FC are individuals and groups with vested interests using the conscience debate to pursue political gain; these parties have also been accused of using the conscience debate to intimidate and bully practitioners to comply with their personal or group ideologies. For example, consider the acrimonious issue of termination of pregnancy: some sources in the 1960s advocated for FC for abortion providers who defied the existing law and SOC at the time, yet some of these same sources have morphed into principal antagonists against FC for those who oppose the current law which permits such procedures. In 1965, for example, an article entitled “*Free the Doctor*,” published in a prominent Canadian newspaper (*Globe and Mail*), demanded liberalization of the abortion law “to enable doctors to perform their duties according to their conscience and their calling” [98]. After abortion was legalized in Canada, however, this same erstwhile public defender of FC advocated on the same issue that all public hospitals should be denied any choice on this issue for any reason-conscience or otherwise [98]. Some agree with conscience choice only to the degree that the choice conforms to their own agenda-the antithesis of what choice actually is. This type of apparent inconsistency has led to suspicions that the issue for some is not FC at all, but of using whatever means necessary to achieve their own agenda.

## 3. Making Decisions in the Face of Ethical Collision 

In light of opposing viewpoints regarding the legitimacy of FC, many physicians find themselves at a moral impasse. Does FC legislation promote discrimination against patient interests and undermine the foundations of modern medicine [57], or are FC declarations integral to ethical healthcare? And more practically, how should individual physicians proceed when faced with ethical situations in which they are called upon to act against their beliefs and their judgment?

Prior to the 1960s, physicians routinely turned to specific codes of ethics as a starting point when faced with ethical dilemmas. For many centuries, the medical community ascribed credence to the venerable Hippocratic Oath, or related ethical principles, as universal points of ethical reference. However, with changes in social mores in the latter aspect of the 20th century, escalating criticism mounted against the Hippocratic tradition, claiming the vows represented a paternalistic “doctor knows best” approach to medicine [78]. This oath was consequently rejected by various administrative bodies, with the assertion by some leading ethicists that physicians who refuse to break their Hippocratic oath are patriarchal or even “genuinely wicked” [99, 100].

### 3.1. Contemporary Codes of Ethical Conduct

Following the demise of the Hippocratic Oath as the ethical standard in medical practice, no single or consistent normative ethical standard has been established to take its place. Currently, there are regional ethical codes of behavior as well as ethical principles inculcated into the hearts and minds of medical trainees by their educational institutions. Such ethical standards have sometimes received diverse interpretations in practical settings.

Regional and international codes of ethics often originate from organizations such as district or provincial medical regulators, national bodies such as the Canadian Medical Association (CMA), and groups such as the World Medical Association (WMA). The CMA Code of Ethics, for example, contains 54 statements relating to physicians' fundamental responsibilities to patients, to society, to the profession, and to themselves [101]. The WMA Code of Medical Ethics offers 22 duties of physicians in relation to clinical practice, to patients, and to colleagues [102]. These statements are instructive in helping an independent physician make ethical decisions and they serve as guiding principles in situations that cause a health professional to encounter a conscience dilemma (Table 2). In disciplinary proceedings, such codes can be used as a standard template against which to measure the conduct of an individual health provider.

While such ethical guidelines are useful as general principles, they do not necessarily provide consistency of care between clinicians; interpretations may differ and subsequent courses of action may vary in accordance with diverse opinions about integrity, best interests, and human rights. For example, one physician may refuse to violate his or her beliefs about a particular intervention claiming it would endanger professional integrity, while another physician may experience no internal disquiet or angst over performing the same intervention—either because he or she does not hold convictions against such procedures, or because he or she is convinced that acceding to patient requests is fundamental to professional integrity. Some argue, in fact, that professional integrity may require the repression of the practitioner's personal human rights [75, 103]. These differences highlight that there are varied interpretations of medical ethics—a reality to be expected in a professional vocation with immense moral and ethical responsibility [87]. Some have argued that within a cultural milieu of plurality, diversity should be tolerated and even celebrated; from this perspective, it would seem consistent that unilateral dictates to denigrate one set of beliefs over another would be frowned upon.

There has also been the introduction of another set of care standards issued by professional societies of specialists that do not have disciplinary or regulatory authority, but which have subtle impact and can be used by regulators in proceedings against an objecting physician. These professional societies frequently claim to be the official voice for their specialty, but in reality they are only accountable to their members. The proposed SOC pronouncements and position statements by such groups are subject to influence by various determinants including vested interests and ideology. In addition, disease-specific advocacy organizations, such as the hypothetical “Osteoporosis Foundation” or the “Depression Society,” often receive funding and support from corporations manufacturing therapies for these diseases. These same advocacy organizations, however, often provide guidelines for care and disseminate pronouncements about how ethical practitioners should counsel individuals diagnosed with these specific diseases.

Finally, various contemporary ethical principles routinely provided to students in medical school training require some measure of ongoing scrutiny. These promoted ideals include values such as beneficence, tolerance, nonmaleficence, nonpaternalism, professionalism, and justice. A major criticism of some of these tenets, however, is that they can be vague, potentially duplicitous, and open to mutually exclusive interpretations [22]. For example, while tolerance of others may be a noble perspective in theory, any sincere disagreement or presentation of an opposing perspective may be characterized as intolerant. With concern about being labeled intolerant, some health providers may be reluctant to challenge poor health choices and then acquiesce to suboptimal courses of action. In essence, alleging intolerance is an effective way to preclude intelligent inquiry and to dismiss honest critique.

### 3.2. Considerations in Ethical Decision-Making

In light of the fact that modern ethical principles do not address specific medical procedures and can be interpreted in many ways, how then are physicians and other healthcare providers to make challenging decisions in situations of ethical distress?

First, we contend that issues of ethical collision should be openly acknowledged and respectfully discussed between professionals and patients. It is important for healthcare providers to disclose their convictions rather than concealing them when considering a course of action they feel is unwise [86]. Failure to disclose the rationale for professional conscience decisions may leave patients confused, in a quandary, and perhaps feeling rejected for the evident disagreement. It is important for patients to be made aware that refusal to provide the requested course of action does not represent the physician's revulsion for the person requesting the service but rather a sincere concern about how the act itself may—from the practitioner's perspective—be unsuitable, imprudent, unethical, or harmful [10]. Furthermore, some critics suggest that acquiescence by the practitioner without being forthright may facilitate guilt and shame for the health provider [37, 104].

**Figure 1:**
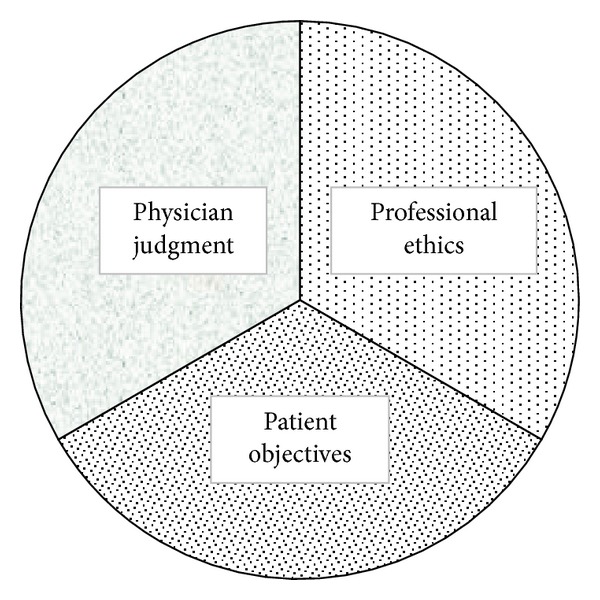
Essential determinants of ethical decision-making.

In order to systematically explore an ethical course of action, it may be useful to consider three components of medical decision-making in light of the case—the patient's objectives, the physician's judgment, and professional ethics (Figure 1). A foundational component of ethical decision-making is an introspective assessment and perspicacious understanding of the ethical values guiding one's decisions. These internal constructs, formed and reformed over the physician's life are crucial in guiding decision-making. It is important that health providers develop insight into their own individual values, the origin of such values, and the way in which these values influence their decision-making.

Inherent in this process is to recognize (i) which values guiding their conscience are deeply held standards, (ii) which represent habitual patterns from socialization, and (iii) which are mere personal preferences. There is a continuum when determining the ethical validity of certain choices and the willingness to be involved in facilitating such choices—the continuum may end with “contrary to” but begins with “not as desirable as.” In order to prepare for situations of ethical collision and to decide on a clinical response, it is important for practitioners to understand their own inherent moral and ethical compass. This process aids in critically assessing whether reservations or oppositions to a medical course of action are justified and can potentially allow for revision or modification.

In all clinical situations, it is vital that a physician patiently and humbly seeks to be empathetic and to understand patient objectives and beliefs in a nonjudgmental manner. The importance of thoroughly understanding patient requests and beliefs cannot be overemphasized. Many physician-patient conflicts can be avoided if both parties understand each other's guiding rationale. Unfortunately, many physicians tend to be burdened by time constraints and this pillar in ethical decision-making can sometimes be neglected.

Finally, an understanding and appreciation of the ethical standards embraced by professional associations is an essential component in ethical decision-making for health providers. For example, in Canada, physicians should be cognizant of the Canadian Medical Association's code of ethics as discussed earlier [101].

### 3.3. Compelled against One's Conscience

Consider a hypothetical case in which an administrator overrules a resident's empathetic decision to resuscitate a developmentally-disabled homeless patient. It is incongruous to assume that compassionate nurses, paramedics, and the resident staff would not have difficulty as a result of seeing a patient denied care. Before implementing any widespread policy to control physician behavior, it is important to consider the impact of unilaterally coercing physicians to comply with authoritarian dictates on all stakeholders within the healthcare system. Moral residue has been described as “that which each of us carries with us from those times in our lives when in the face of moral distress we have seriously compromised ourselves or allowed ourselves to be compromised” [105]. There is emerging attention to the potential personal consequences “when there is incoherence between one's beliefs and values and one's actions” [105].

To the authors' knowledge, no quantitative research exists to date to measure the impact of moral residue or to objectively determine the outcome of violating personal conscience in medical practice. Recent anecdotal evidence, however, suggests that failure to act in accordance with deeply-held beliefs in times of “moral distress” may have damaging sequelae. There is increasing discussion about the concept of moral trauma or “moral injury.” This latter term refers to consequences resulting from “perpetrating, failing to prevent, bearing witness to, or learning about acts that transgress deeply held moral beliefs or expectations” [106]. Although it is not known how the symptoms of moral injury will present over time, there is concern that the consequent psychological and emotional strain may have a detrimental impact on the essence of personhood. As an individual's moral framework may constitute a fundamental component of their identity, coercion to engage in behavior that violates their moral code may represent an assault on their moral ecosystem and a violation of personal integrity that threatens their essential humanity [12]. In military situations, for example, moral injury can be associated with serious and ongoing alienation, intense shame, and sustained distress [107].

Preliminary evidence gleaned from study of various types of health professionals is noteworthy. In addition to immediate feelings including anger, resentment, guilt, frustration, sorrow, and powerlessness when faced with serious moral distress [42], the recent literature has begun to describe anecdotal long-term sequelae of the associated moral trauma inherent with ethical distress that sometimes results in conscience violation. Health professionals have been noted over long-term observation to display emotional dysregulation and experience problems including job dissatisfaction, abandonment of their profession, burnout, feelings of inadequacy, relational challenges, and alterations in patient care [108–112]. Undoubtedly, observational research to quantify impact of violating personal conscience is challenging due to confounders including personality differences, support systems, and healthcare-provider confidentiality. It is possible, however, that health professionals who compromise their conscience and violate their moral compass may be casualties of any ruling that disrespects conscience freedom.

What impact does violation of conscience have on integrity of conscience? Research involving other professions suggests that stifled consciences may lead to permanently “seared” consciences [11]. Just like the death camps of World War II, where the perpetrators of horrific crimes including some doctors were socialized into disassociating their conscience from their conduct, so also can other physicians be subtly compelled to become skilled technicians submitting to authority [11]. Some doctors in South Africa, for example, succumbed to hierarchical pressures to condone ongoing acts of state-sanctioned violence under the Apartheid regime [113].

Patients and society will also face the effects of physician moral dissatisfaction. If practitioners become increasingly subservient technicians, rather than self-regulated medical advisors, patients will no longer able to trust that a physician's advice is based on a personal assessment of what is best for the patient. Recipients of health care will be left to decipher medical recommendations based on what they assume to be the underlying purpose of the counsel. In addition, physicians will become increasingly dependent on authorities and regulators (who may be influenced by vested interests) to dictate what they can or cannot do. As mentioned, physicians who act as technicians at the beckoning of the state have carried out many atrocities [68]. Certainly, any society that encourages obedience without questioning not only places all of humanity in a precarious position but also limits the freedom of healthcare institutions throughout society [10, 11]. In addition, some clinicians attribute the marked pattern of declining physician morale in some measure to the fact that medical practitioners are no longer self-regulated, but are increasingly subject to and regulated by administrators who themselves have little to no clinical responsibilities [22, 114].

Physicians refusing to comply with given guidelines may face a difficult choice: (i) finding a surreptitious means of avoiding uncomfortable actions (i.e., calling in sick, refusing to accept certain patients, changing shifts), or (ii) accepting penalization in order to save their personal integrity [10]. Should healthcare providers and trainees acting from a perspective of conscience face penalties such as rejection from medical training, loss of privileges to practice within an institution, or even a requirement to surrender their medical license if their chosen course of action is in disagreement with a patient or medical regulator?

Most ethical questions involve subjective judgment and often cannot be answered by “empirical testing or any other comprehensive doctrine for distinguishing right from wrong” [115]. Accordingly, if it is impossible to objectively determine that either of two ethical poles is right, both sides of this argument must concede that there is at least some possibility that opponents may be right, leaving no legitimate grounds on which to punish them [115]. Based on respect for diversity, legal and policy precedents, ethical uncertainty, and the potential impact on individual medical professionals and society as a whole, we conclude that it is intolerant, illegitimate, and immoral to punish health providers who act based on deeply-held conscience perspectives about what they believe is best for patients.

## 4. Broader Perspectives on Freedom of Conscience

The authoritarian stance of coercing health professionals to do what they sincerely believe is wrong appears to be unsupported on many fronts. The Canadian Medical Association Code of Ethics Article 7, for example, charges physicians with the responsibility to refuse any medical participation that will undermine their professional integrity [101]. This article and many others in the Code of Ethics (explored in Table 2) emphasize that a physician possesses the responsibility of not only upholding the patients' best interests, but also the responsibility to maintain his or her own personal integrity. Facilitating a clinical course of action that the health provider sincerely deems to be ill-advised, unethical, or against the patient's best interests may compromise the integrity of the professional role and may violate fundamental tenets of such ethical codes. Furthermore, the WMA further emphasizes the importance of physicians' “independent professional judgement” and “moral independence” [102], and claims that physician independence is a fundamental component of acting ethically in the patient's best interest.

Some freedom of conscience opponents contend, on the other hand, that it is both arrogant and paternalistic for a physician to consider that he or she knows what is in the patient's best interests and they assert that a refusal to accede to patient requests represents an imposition of values [57, 69, 70]. Conscience supporters rebut this claim by suggesting that the practice of medicine is predicated on the reality that a patient consults a health provider seeking advice and counsel to the best of the practitioner's ability and skill—just as an individual seeking professional advice from a lawyer is seeking counsel to the best of the advocate's knowledge, wisdom, experience, and ability. It would appear to be ethically problematic for a lawyer to facilitate a course of action he or she deems seriously harmful to the client. While it is true that the actions of any professional are not necessarily correct objectively, they are deemed to be the best representation of the ability of that individual who has been granted the privilege of acting as a professional.

Medical practice is also a fundamentally human and personal enterprise, an ideal that is compromised when the profession is subservient to the state or overarching social and professional dictates. Furthermore, medical professionals are not simply service providers or therapy vendors, but professionals using judgment, wisdom, and decision making-nonobjective concepts that will certainly be in error at times. The fact that the privilege of prescribing medication is restricted to physician judgment, not simply patient request, for example, is representative of the respect given to the wisdom and experience of the professional rather than leaving this decision solely to the patient's judgment.

Although the medical community is a self-governing profession, it is also subject to the law with adherence to national and international charters. Canadian citizens, for example, are protected under the Canadian Charter of Rights and Freedoms. This Charter specifically states that Canadians enjoy fundamental FC [116], a perspective that has been upheld by legal rulings in the Supreme Court of Canada. In regards to FC, it is noteworthy that in 1985, for example, Chief Justice Brian Dickson established a legal precedent upholding the freedom of Canadians to refuse to be coerced or constrained to act, or to refrain from acting, in a manner contrary to their volition [117]. When discussing freedoms, the justice wrote: “Freedom can primarily be characterized by the absence of coercion or constraint. If a person is compelled by the State or the will of another to a course of action or inaction which he would not otherwise have chosen, he is not acting of his own volition and he cannot be said to be truly free” [117].

Although differing interpretations exist, some understand this judgment to suggest that no Canadian is to be compelled to perform an action that is contrary to his or her beliefs or conscience, as long as it is within reasonable civil limits and does not jeopardize the freedoms of others. Some others, however, contend that while freedom is a noble pursuit, it is legitimate in some situations to constrain absolute freedom in order to achieve a higher individual or public good—such as the situation of forced confinement for someone threatening to harm others or self. As a result of differing perspectives and interpretations of the meaning of freedom, an increasingly common challenge facing the justice system is to consistently find appropriate balance in the tension between individual rights and the perceived greater personal or public good.

As well as the existence of country-specific charters, the United Nations (UN) has crafted a Universal Declaration of Human Rights, which appears to add another layer of support in protecting a physicians' right to a free conscience. Article 18 explicitly states that “everyone has the right to freedom of thought, conscience, and religion” [118]. This article and others contained in the UN document expound on the fundamental rights and responsibilities of all humans, including practicing physicians.

Regardless of the fact that charters and precedents may support conscience rights, many practitioners still feel compelled to violate their own conscience in some clinical situations. While charters may offer theoretical refuge, some clinicians conclude that proclamations hold little sway within regional medical communities [7]. In the face of enormous pressure and sometimes ethical anguish, it is important for professionals to also consider the potentially damaging sequelae of acting against their conscience, a concern that has unfortunately been for the most part neglected in the conscience debate.

### 4.1. Other Considerations about Freedom of Conscience

Conscientious objections today are plagued by shifting lines in the sand—while a medical act may be frowned upon one day, legislative or social changes may result in the condoning of the same act a short while later. Furthermore, policies often conflict between localities. This pattern is currently evident in the protocol surrounding end of life interventions—some jurisdictions are vehemently opposed to euthanasia while other locales support this practice. Similarly, female genital mutilation is considered abhorrent in many jurisdictions and cultures, but is routinely practiced in other areas and among some cultures. Does something become good or evil based on what authorities decide or what geographical area it is undertaken? It is doubtful whether a physician's conscience should be dictated by geography or the whims of legislators or judges in a given region.

Patient autonomy and physician autonomy are not mutually exclusive and are not competing ideals. In an era of alleged respect for personal autonomy and independence, denial of conscience rights is a repudiation of physician autonomy. Rather than the physician presenting patients with choices and recommendations with informed counsel and respecting the patient's right to make autonomous decisions based on informed consent, removal of FC relegates physicians to become service providers subordinate to patient and regulatory demands. Rather than respect for patient autonomy in the physician-patient relationship, such a trend moves medicine into the realm of patient “sovereignty”—a forfeiting of physician autonomy in which health professionals are expected to separate their professional acts from their personal values.

Finally, much attention has been applied to the sacrosanct and confidential physician-patient relationship. It is questionable whether those outside the profession who are not directly involved in unique patient-physician encounters should be overarching commanders in dictating the outcome of such interactions. While many contend that decisions regarding certain ethical matters should remain an issue between a patient and their doctor, denial of FC eliminates this construct completely by making such interactions ultimately an issue between a patient and regulators.

### 4.2. Additional Concerns about Conscience Freedom Legislation

While most patients expect their health professional to be ethically-minded, knowledgeable, honorable, and compassionate, it is plausible that unregulated FC clauses could become a “rule that knows no bounds” [70]. Certain common concerns with broad FC declarations have been voiced by both critics and supporters of conscience rights legislation [1, 10, 27, 55, 57]. Exploitation of liberties and fallibility of conscience are two main issues that have been raised as potential challenges.

A universal FC clause may facilitate behaviour considered by most to be problematic or profoundly inconvenient under the guise of “conscience rights.” It is conceivable that physicians could refuse to see or examine patients of a particular gender or lifestyle, with specific types of medical conditions, or choose to miss work on cultural or religious days [56]. Furthermore, some physicians may decide not to provide care to seniors past a certain age, to decline the acceptance of patients with complex health problems, or to refuse to learn about sexually transmitted diseases because of personal prejudices [119]. In fact, there are reports of medical students from one religious group refusing to learn about alcohol-related diseases or to assess and treat members of the opposite sex [65]. A physician-in-training who, allegedly based on conscience, refuses to learn how to care for patients within a certain demographic or with selected medical conditions, poses a significant impediment to medical education [87].

This “double-edged sword” aspect of the FC issue extends to behavior or actions considered abhorrent or deplorable by social standards and highlights an apparent inconsistency among supporters of FC. There is genuine apprehension that any formalized FC policy might facilitate tolerance of repugnant behavior that is not socially acceptable but which serves the personal conscience of individual practitioners. Concerns on this matter have been expressed about certain choices by health providers surrounding issues including female genital mutilation, virginity certificates, or the refusal to resuscitate disabled newborns and elderly Alzheimer's patients. Just because an individual or group of health providers from a particular perspective feel compelled by conscience to support or refuse a medical practice does not necessarily translate into support from FC advocates. Ultimately, many FC supporters acknowledge the inconsistency and grant that conscientious decisions within a civilized society must have delineated boundaries.

It is well recognized that just as sincere regulators can be sincerely misguided, sincere individual practitioners can be sincerely misguided. Just as governments and administrators are not infrequently misguided in their decisions, individual well-meaning professionals may also be misguided in their judgments, even with good intention. Both individual as well as collective conscience can be very subjective, fallible, and heavily influenced by disordered reasoning, misinformation, peer influence, and societal or cultural pressures. Such pragmatic concerns about FC legislation highlight the challenge for any group functioning within an environment without a normative ethic or with a plurality of ethical perspectives based on different fundamental values.

Various suggestions have been put forth to address such concerns. Physician accountability is absolutely required to secure public and patient safety and to preserve the integrity of the profession. Accordingly, in the absence of a normative ethic, a delicate balance of regulation and respect for individual freedom is necessary [22]. It is our view that professional bodies and legislators should fulfill their primary role of protecting the public good within reasonable boundaries but should concomitantly establish some overt measure to demonstrate tolerance towards conscientious, competent physicians who demonstrate disparate views on the continuum of ethical diversity [56].

Some have suggested that open, respectful discussion between colleagues of diverse perspectives may help serve as a suitable safety net to cut through erroneous reasoning, emotional tension, and/or peer pressures [10]. This reasoning suggests that honest exploration of the issues would help healthcare workers develop realistic approaches to deal with conscientious objections [56]. Although well-intentioned, the current culture of medicine does not necessarily always foster or condone open discussion [120]. A common portrayal of conscientious objectors depicts such healthcare workers as intellectually challenged religious fanatics who impose their personal values on patients and dogmatically refuse to provide patients with legal, well-accepted medical treatments. Opponents of conscience rights are sometimes quick to further stereotype such conscientious objectors as obscure outliers with philosophies and views contrary to mainstream evidence-based ethical care. As a result, some contend that while openness to thoughtful discussion of conscience issues should be encouraged, the only option that will secure the human rights of minorities at this time is FC legislation. While this defensive measure may not be ideal, it may be required to prevent tyranny in selected situations.

### 4.3. Suggested Approach When Considering Situations of Ethical Tension

Suggested guiding principles for healthcare providers to demonstrate respect for patients while maintaining conscience and personal integrity are offered for consideration in Table 3. An actual case study is then presented which illustrates some of the practical realities of enacting FC in a clinical context and highlights some of the professional issues associated with divergent perspectives on common medical interventions.

## 5. Case Study

While consulting on the cases of two young women with cerebrovascular events following commencement of the birth control pill (BCP), a physician became aware of emerging information presented in the medical literature related to this medication. After much consideration, the physician eventually made a conscience decision to no longer dispense oral contraception (OC). This choice was not in keeping with the current SOC and resulted in several uncomfortable situations with patients and colleagues.

In coming to this decision, this medical professional initially reviewed the medical literature related to hormonal contraception. It was found that most BCP research and the associated knowledge translation appeared to be funded by vested interests—industries associated with OC (oral contraception), as well as groups and professional associations with ties or receiving funding from contraceptive manufacturers. With extensive literature confirming the enormous influence of industry on research outcomes [93, 121–123], and multibillion dollar settlements against various major pharmaceutical companies for egregious wrongdoing [124], the integrity of some of the alleged findings in the industry-sponsored reports was questioned. Furthermore, on detailed review of various research publications, numerous adverse findings relating to individual and public health were evident regarding BCP use. A small sample of recent references to summarize selected concerns includes the following.

- The BCP is a human carcinogen in women [125–127], in men [128] (through environmental contamination), and in offspring [129] (through vertical transmission).
- The BCP significantly increases the risk of cardiovascular events [130], hypertension [131, 132], and cerebrovascular disease [133].
- The BCP is a significant determinant of diminished and irreversible female sexual dysfunction [134, 135].
- The BCP exerts an adverse effect on mood in some women [136, 137].
- The BCP is a widespread and escalating endocrine disrupting contaminant in the ecosystem and domestic water supply [128, 138, 139].
- Some BCPs increase the risk of adverse birth outcomes and allergy in offspring of users [140, 141].

With the eventual decision to no longer prescribe the pill, some challenges ensued. As the BCP is the most common method used for fertility regulation, many of the physician's patients were already hormonal contraceptive users. Furthermore, while taking evening and night call for other practitioners, awkward situations arose as the physician interacted with colleagues' patients who requested BCP refill prescriptions. When the reasons were presented to patients along with other family planning options, an array of responses ensued. Most people politely listened to the information; some were grateful and chose to reconsider BCP use, several were decidedly not interested in the information, and a few conveyed displeasure. All were expressly aware they could acquire a BCP prescription refill from other physicians. Just the same, most patients were inconvenienced and some were disgruntled by the refusal to provide a prescription. A few patients were surprised to hear about such risks and wondered why they had not been informed previously. A few, including a medical student, suggested the information was not true, and accused the practitioner of trying to impose religious beliefs on patients.

The physician's decision to not prescribe the BCP was generally received unsympathetically by colleagues. This disapproval was sometimes reflected by direct responses including: “It is so archaic and out of step with reality and modern medicine to not support hormonal contraception,” and “Modern clinical practice guidelines include dispensing birth control pills. If you cannot abide by the guidelines, then do not be a doctor.” Interpersonal professional relationships with a couple of colleagues became uncomfortable as they were inconvenienced by the refusal to refill BCP prescriptions.

When the physician's rationale was directly communicated to colleagues, most expressed initial skepticism of the supposed scientific concerns. When provided with references and medical literature, these colleagues were generally surprised and had minimal refutation other than responding that it was necessary to continue prescribing OC because of patient demand, and that patients had a right to make their own decisions. The physician expressed concern that most patients were not apprised of the aforementioned risks and thus no informed consent was obtained. If physicians were not themselves aware of the risks, it was certain they were not communicating such risks to patients. Furthermore, beyond the patient's right to put herself at risk, hormonal contamination of the water supply with ethinyl estradiol exposes the unsuspecting public to health risks, evidenced by the scale of prostate cancer risk in areas of high BCP use as recently discussed in the *British Medical Journal *[128]. While colleagues were generally unaware of emerging options for family planning discussed in the literature including new high-tech fertility monitors [142, 143], most decidedly lacked interest in discussion of such options.

In a subsequent election campaign, the regional government where the physician practised medicine unexpectedly announced that physician conscience rights—specifically the refusal to prescribe the BCP—would not be tolerated [144]. This pronouncement raised the issue of whether the physician's ability to practice conscientious medicine would be compromised by legal regulation. In addition, it became evident that some other health providers and medical trainees in other regions of the country had been chastised or disciplined by regulators for refusing to prescribe the BCP. These situations introduced the question of the role of medical and state officials in protecting the public good and whether such authorities have the knowledge and competence to always do what is best for healthcare.

Like many jurisdictions, the U.K. General Medical Council for example, continually updates and enforces a code (the UK document is entitled “Good Medical Practice” [145]) which sets forth appropriate physician behavior in their mandate of “regulating doctors, ensuring good medical practice.” Despite this type of stringent regulation in most locales, however, the widespread and atrocious rates of persistent iatrogenic morbidity and mortality associated with many common and approved medical interventions [16, 146–152] confirm that perhaps some of what is sanctioned by regulatory bodies is routinely harmful to many patients. Furthermore, recent literature also confirms that many standard medical guidelines are heavily influenced by vested interests [96, 153–155] and are dated due to the slow rate of knowledge translation [92–94]. These observations account for many of the not infrequent flip-flops in recommended medical interventions, such as the HRT (hormone replacement therapy) debacle [96].

After much consideration and study, the physician concluded that what is considered acceptable or “good” medical practice by regulatory bodies is not always objectively “good” for patients. In an age of evidence-based medicine, credible outcomes and “evidence” are the markers of good medical practice, rather than the subjective perspectives of regulators. The practitioner determined that guidelines within the profession are sometimes not trustworthy, and with the enormous influence of industry on these pronouncements, they are, at times, unethical [96, 121, 122, 155]. In addition, it became evident that throughout medical history, recognized and celebrated advancements in medical practice have frequently occurred because conscientious practitioners refused to comply with the status quo [153]. Considerable discussion with respected colleagues and scientists ensued to confirm the legitimacy and accuracy of the expressed concerns regarding the BCP and the state of contemporary medical practice. The physician concluded that it is misguided for medical authorities to diminish the role and importance of personal conscience and moral awareness in medical practice.

Every clinical judgment is configured within a premise of conscience—the premise that a physician ought to provide the best available treatment, and that it would be unethical not to deliberately refuse to do otherwise. It was from this stance that a conscience decision was enacted. As such, after studying the scientific literature and consulting with respected experts, the physician concluded that with effective and safer alternatives readily available, dispensing hormonal contraception routinely was perhaps not in the best interests of patients or society as it is apparently endangering to personal and public health, destructive to the environment, and potentially harmful to wildlife. The physician in this case study made a conscience decision based on moral precepts of “doing the right thing” to no longer dispense the BCP. Patients' need for fertility regulation was attended by providing comprehensive information about all family planning options and recommending approaches that the physician sincerely felt were optimal.

## 6. Conclusion

The dilemma of diversity is not new. Diversity of ethics and morals is the natural consequence of a culture that facilitates freedom of thought, independent thinking, and moral autonomy. Although such precepts as liberty of thought and action in all domains may sound reasonable, many philosophers including 19th century authors Friedrich Nietzsche in Germany and Fyodor Dostoevsky in Russia have cautioned about the typical sequelae of such liberty. These noted thinkers have suggested that with the passage of time, freedom of diversity might be anarchic, destructive, impossible to sustain, and something that has to be constrained in order for people and cultures to thrive [156, 157]. Yet, despite historical concerns, our contemporary culture currently claims to respect and celebrate freedom of thought and diversity, an inclusive perspective which is generating escalating angst and conflicting responses from within the medical community. Can contemporary medical culture tolerate nonuniformity of values and thrive in the face of conflict on basic issues including definitions of what constitutes human life?

As our society becomes increasingly multicultural and diverse in the marketplace of ideas and in the everyday domains of contemporary western life, it is uncertain whether our culture can sustain a tolerance and respect for the potentially polarizing views represented by escalating diversity. In the medical community specifically, the shift away from the definitive normative ethic of the Hippocratic Oath to the modifiable and equivocal “codes of ethical conduct” in the 1960s may have initiated a more significant transition than is generally recognized. With dissimilar and sometimes mutually exclusive interpretations of what is good, prudent, and necessary for patient care, healthcare providers will inevitably face ongoing challenges with moral and ethical dilemmas. Such diversity raises various questions. With the divisive and sometimes acrimonious exchanges on various ethical issues that take place, will regulatory authorities sense a threat to the homeostasis of the healthcare community and move to establish an authoritarian approach to constrain ethical diversity? With plurality of thought on various healthcare issues, which faction within the medical or regulatory community has the moral and scientific authority to decide upon foundational pillars and clinical directives of any new normative ethic?

Yet, there is also legitimate concern that enforced uniformity and allegiance to the dictates of any authority, thus coercing health providers to abandon diversity and conscience in order to accede to fluctuating social norms and patient demands, has the potential to threaten individual integrity and, in some situations, to endanger society. Furthermore, compulsion cannot eliminate personal moral awareness, and coerced participation in morally repugnant acts imposes “unnatural” motivation on the healthcare provider [101]. Consideration of the moral, emotional, and psychological trauma which may be done to individuals compelled to act against conscience is an important part of this discussion and warrants careful study. To date, analyses of the impact of coerced involvement have tended to focus on the outcomes for healthcare systems and recipients rather than for providers; a notable deficiency considering the importance of medical professionals as key stakeholders in providing sustained care within the healthcare system.

Many essential questions on this issue, for example, remain unanswered. Does repeated moral distress lead to damaging moral injury with attendant sequelae? Is denial of conscience a pathway to nullification or euthanization of conscience? What is the impact of moral stress on delivery of patient care and the physician-patient relationship? With high rates of burnout and about one-quarter of physicians already expressing that they feel depressed [158], can healthcare systems afford to have increasing numbers of walking-wounded among their healthcare providers?

After considering the emerging literature and the myriad of opinions on all sides of the equation, it is proposed here that abolition of conscience freedom is not apposite within contemporary healthcare. In the interests of society, the profession, and the advancement of medicine, it seems misguided for authorities and regulators to introduce a draconian policy of coercing clinicians to set aside iconoclastic ideas, to avoid scrutiny of the status quo, and to suspend professional judgment on various fundamental health issues. Such a policy of intolerance towards individual freedoms and creativity, often engineered by individuals far removed from the practice of clinical medicine, displays a lack of respect for the competence, ability, ingenuity, and integrity of health professionals, and has the potential to stifle medical progress and to adversely affect physician morale. History has repeatedly established the progressive role of thoughtful dissent in the delivery of healthcare. It is therefore suggested that a judicious tension of individual freedom and competent regulation within accepted societal boundaries is required to facilitate a vibrant and progressing professional environment. It is apparent, however, that some governments and medical regulators are entertaining the idea of adopting an authoritarian role and purging liberty of conscience from healthcare professionals.

In a recent election campaign in Canada, an intense debate unfolded on the healthcare conscience issue at which time the government leader, challenging the principle of conscience freedom, stated “when people take on professional responsibilities, I expect them to be able to meet those professional responsibilities” [144]. It will be a noteworthy and significant day for individual practitioners, for the medical profession, for individual patients, and for society as a whole when we demand a preparedness to do what one believes to be unethical, wrong, or evil as a prerequisite professional responsibility in order to join the medical community. It will be a sobering moment, indeed, when a willingness to capitulate to regulatory demand becomes a more important and established value in the medical community than integrity of character and an unwavering resolve to do what is good. It will be a paradoxical state when we exhort doctors to “Do no harm” but simultaneously compel them to do what they believe is harmful—as long as a patient requests it or an authority demands it.

## Figures and Tables

**Table 1:** Examples of clinical situations that may result in ethical tension or conscientious refusal.

Dilemma	Situation
(i) Government pressures physician to perform punitive amputation	Orthopedic surgeon told by Afghani government officials to amputate a healthy man's leg as a punishment for theft [13].
(ii) Physician pressured to perform CPR	In a case situation consistently deemed medically futile, a clinician refuses to prolong dying, squander resources, and extend patient suffering by repeatedly commencing CPR [14].
(iii) Peer pressure for physician to conform to standard of care guidelines	A doctor is derided for using evidence-based nutritional and environmental interventions where such therapies deviate from standard clinical practice [15, 16].
(iv) Patient requests physician to complete paperwork so parents can travel for cultural ceremony	Parent requests official approval from a physician for their daughter to travel to Africa in order to undergo a ritual female genital mutilation ceremony [17].
(v) Physician asked for advice about suitability of abortion	Patients seek advice from a rural physician on suitability and wisdom of having an abortion after discovering that the developing fetus has cystic fibrosis [18].
(vi) Physician asked to determine fetal gender	Request that the physician determine fetal gender at 12 weeks gestation with the expressed aim of choosing female feticide if the fetus is not male [19].
(vii) Patient request for assisted suicide	An elderly patient adamantly requests that a physician prescribe a lethal dose of sedation [20].
(viii) Peer pressure to increase hospital efficiency at the cost of patient care	A physician is unable to provide optimal care for seniors with severe dementia as a result of explicit institutional economic constraints [21].
(ix) Young patient requests tubal ligation	Following the delivery of a stillborn child, a 19 year old with no live children determinedly requests an irreversible tubal ligation procedure [22].
(x) Patient request for genital reconstruction	Adult female requests a re-infibulation procedure (reconstruction of ceremonially cut female genitalia) following vaginal childbirth [23, 24].
(xi) Patient demands narcotic analgesia	Physician is suspicious of narcotic abuse with the patient [25].
(xii) Parents of child refuse consent for life-saving blood transfusion	Physician considers legal measures to save the life of the child through blood replacement [26].
(xiii) Parents of young woman request virginity certificate	Based on personal moral beliefs, the clinician refuses to exam the hymen of the young woman-despite explicit consent from the young woman herself.
(xiv) Patient demands respect for personal autonomy in choice of physician	A pregnant woman refuses emergency obstetrical care based on the clinician's gender and race. She demands referral to a female physician.
(xv) Patient requests distortion of truth	A terrified immigrant woman implores her family physician to lie to her husband regarding the nature of a previous surreptitious medical visit.

**Table 2:** Excerpts from the Canadian Medical Association [101] and World Medical Association [102] Code of ethics.

(i) Consider first the well-being of the patient (CMA # 1)	(i) A physician shall always exercise his/her independent professional judgment and maintain the highest standards of professional conduct (WMA # 1.1)
(ii) Practise the art and science of medicine competently, with integrity and without impairment (CMA # 5)	(ii) A physician shall be dedicated to providing competent medical service in full professional and moral independence, with compassion and respect for human dignity (WMA # 1.4)
(iii) Resist any influence that could undermine your professional integrity (CMA # 7)	(iii) A physician shall respect the right and preferences of patients, colleagues, and other health professionals (WMA # 1.7)
(iv) Refuse to participate in or support practices that violate basic human rights (CMA # 9)	(iv) A physician shall act in the patient's best interest when providing medical care (WMA # 2.2)
(v) Inform your patient when your personal values would influence the recommendation or practice of any medical procedure that the patient needs or wants (CMA # 12)	(v) A physician shall give emergency care as a humanitarian duty unless he/she is assured that others are willing and able to give such care (WMA # 2.5)
(vi) In providing medical service, do not discriminate against any patient on such grounds as age, gender, marital status, medical conditions, national or ethical origin, physical or mental disability, political affiliation, race, religion, sexual orientation, or socioeconomic status (CMA # 17)	

**Table 3:** A suggested approach for healthcare providers when facing conscience dilemmas.

(i) Be an excellent MD in competence, knowledge, compassion, and relationship with patients.	
(ii) Avoid emotional manipulation; always provide the complete truth and comprehensive information.	
(iii) Always do what you believe to be right and best for the patient.	
(iv) Prepare patients early on in the relationship for any perspectives that may be at odds with the patient's values.	
(v) Consider referral to appropriate regulatory bodies for patients needing further direction.	
(vi) With sincerity, respectfully explain your perspectives when in disagreement with patients.	
(vii) Respect individual values and ethics but never compromise your personal honor and integrity.	
(viii) Expect that some people will not appreciate you; most will.	
(ix) Continually examine your actions and motivations with humility and secure a means to maintain continued accountability. Respectfully discuss concerns with regulatory bodies as appropriate.	
(x) Always approach medical authorities with respect and avoid insubordination. Refusing to perform an action that is sincerely perceived to be unethical, however, is not insubordination.	
(xi) Obtain advice, and share ideas and concerns with trusted colleagues.	
(xii) Confirm for patients that they have the right to see another health provider.	
